# Sequence tube maps: making graph genomes intuitive to commuters

**DOI:** 10.1093/bioinformatics/btz597

**Published:** 2019-08-01

**Authors:** Wolfgang Beyer, Adam M Novak, Glenn Hickey, Jeffrey Chan, Vanessa Tan, Benedict Paten, Daniel R Zerbino

**Affiliations:** 1 UC Santa Cruz Genomics Institute; 2 Department of Biomolecular Engineering, University of California, Santa Cruz, CA, USA; 3 European Molecular Biology Laboratory, European Bioinformatics Institute, Wellcome Genome Campus, Hinxton, Cambridge, UK

## Abstract

**Motivation:**

Compared to traditional haploid reference genomes, graph genomes are an efficient and compact data structure for storing multiple genomic sequences, for storing polymorphisms or for mapping sequencing reads with greater sensitivity. Further, graphs are well-studied computer science objects that can be efficiently analyzed. However, their adoption in genomic research is slow, in part because of the cognitive difficulty in interpreting graphs.

**Results:**

We present an intuitive graphical representation for graph genomes that re-uses well-honed techniques developed to display public transport networks, and demonstrate it as a web tool.

**Availability and implementation:**

**Code:** https://github.com/vgteam/sequenceTubeMap.

**Demonstration:**

https://vgteam.github.io/sequenceTubeMap/.

**Supplementary information:**

Supplementary data are available at *Bioinformatics* online.

## 1 Introduction

Graph structures have been abundantly used for a wide array of genome sequence analyses, such as *de novo* assembly (Medvedev *et al.*, 2009) or whole genome alignment and variation analysis (Paten *et al.*, 2017), because they offer a flexible and compact way of representing sequences that differ slightly, whether for biological reasons (e.g. polymorphisms) or technical ones (e.g. sequencing errors). In particular, graph genomes allow sequence mappers to take structural variation and polymorphism into account and reduce bias in variant calling (Paten *et al.*, 2017).

Software libraries such as *vg* (Garrison *et al.*, 2018) can efficiently store DNA sequences and compute or extract alignments between them. Its Graph Alignment Map file format can store the alignment of short sequencing reads to a graph. We set out to develop an intuitive display to examine the alignments of sequencing reads to a graph genome, as produced by *vg’*s mapper. To our knowledge, there exists no solution to view both structural variation and sequence alignments.

From a technical point of view, genomic graphs are generally isomorphic to a specific subtype of graph called bi-directed graphs (Medvedev *et al.*, 2009). In these graphs, all the edges connected to a node are clearly divided into two sets, often represented as incoming and outgoing edges or else as edges connected to one of two ends of the node. The node thus conceptually has two ends, like the DNA fragments it represents. If entered through one end, a node must be exited through the other. A node thus corresponds to a sequence of nucleotides when entered through one end, and its reverse complement when entered through the other. Each edge is characterized by its direction through either node it connects to, hence is bi-directed. Genomic sequences are thus represented as sequences of nodes connected end to end.

Consequently, many tools of graph theory do not apply perfectly to bi-directed genomic graphs. Current graph genome visualization tools (Nielsen *et al.*, 2009; Wick *et al.*, 2015) generally represent DNA fragments as edges and their connections are collapsed into isotropic nodes. This produces layouts where the orientation of edges along meaningful sequences such as haplotypes are not coordinated, creating paths that zig–zag randomly across the graph’s diagram.

We propose here a graph layout approach for genomic graphs that focuses on maximizing the linearity of selected genomic paths. It is heavily inspired by London’s iconic Tube Map, as designed by Harry Beck in 1931, which was itself inspired from circuit diagrams (Garland, 1994). There exist algorithms to automatically draw such maps (Wolff, 2007), however, for speed and interactivity we opted for heuristics in this implementation.

## 2 Materials and methods

**Fig. 1.**
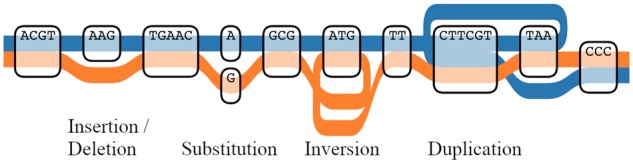
Two genomic sequences represented with a tube map

**Fig. 2.**
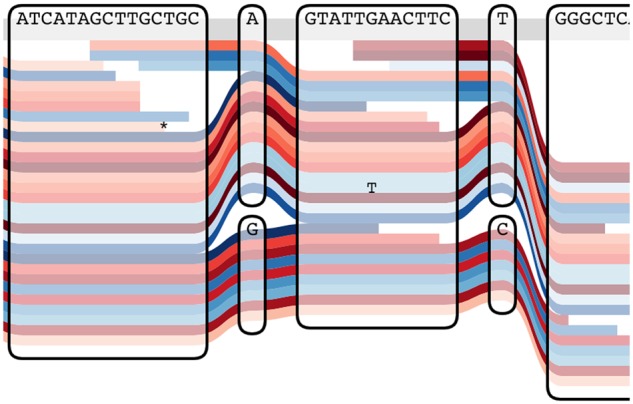
Representing sequencing reads on a graph. The sequence of the reads is only represented on the bases where they differ from the reference

The atomic elements of our representation are bi-directed nodes, drawn as rounded rectangles. The left and right edges of the rectangles are the ends of the nodes, and the direction of an edge incident on a node is thus represented by which side it is attached to. At the top of the rectangle is the sequence which corresponds to traversing the node left to right. Through these nodes we draw out representative paths as colored lines, which can correspond to haplotypes or sequencing reads. A given path can traverse a node an arbitrary number of times, thus representing deletions or duplications. Every time it traverses a node, it can go in one direction or the other, thus representing inversions. The example in Figure 1 illustrates how all types of structural variants can be represented. Optionally, it is possible to mark on the paths the bases where they differ from the reference. Figure 2 illustrates the usefulness of Tube Maps when displaying short reads mapped to a graph genome.

The graph layout algorithm is heuristic and tries to arrange the graph horizontally with the sequence paths running from left to right (or from right to left). In a first pass of the algorithm, all nodes are arranged into horizontal slots. The algorithm walks along one sequence path after another, trying to arrange the unassigned nodes it passes through from left to right. There can be multiple nodes in a single slot, which means these nodes have the same horizontal but different vertical positions. In the second pass, the algorithm passes over each horizontal slot from left to right and lays out its content (the nodes and all sequence paths traversing this slot, whether within a node or not) vertically. This step is greedy, minimizing the sum of the absolute differences of each sequence path’s vertical position compared to the same path’s position in the previous slot.

Our implementation is enhanced by interactive features. For example, hovering above one of the sequences highlights it across its entire length, which can make it easier to follow in the presence of many other sequences. Double clicking on it turns it into the reference sequence for the graph, which is instantly re-arranged (Supplementary Material).

## 3 Results

We implemented SequenceTubeMap, a JavaScript module that displays *vg* files. To visualize a specific *vg* file, it is possible to launch a server which provides the data to SequenceTubeMap. The same visual representation could possibly be adapted to other graph genome formats, provided they can be queried through an API.

We have successfully used SequenceTubeMap to visualize a graph of the 2504 haplotypes of the 1000 Genomes Project (The 1000 Genomes Project Consortium, 2015), as well as reads mapped against it. We provide a tutorial along with the SequenceTubeMap software that describes how user-provided data can be visualized with the tool; sufficiently small files can be uploaded directly to our demonstration instance.

In practice, the ability to readily visualize alignments on a graph genome has proven invaluable in shortening the de-bugging cycle in our work on the *vg* mapping tool. Although the view can get progressively cluttered in the presence of many re-arrangements, it is particularly useful in examining alignment details at junction points or around small structural variants.

## 4 Conclusion

There is a growing need for genomic tools that can handle the complexities of polymorphism and structural variation. This in turn will require researchers to adapt their work to this enriched context, for example to examine alignments and variant calls in polymorphic regions. Intuitive visualization tools such as the sequence tube maps will help them explore and become familiar with graph genomes.

## Supplementary Material

btz597_Supplementary_Material
