# Supplementary material for: Sequence tube maps: making graph genomes intuitive to commuters
Source: Bioinformatics. 2019 Aug 1;35(24):5318–20. doi: 10.1093/bioinformatics/btz597 (PMC6954646; doi:10.1093/bioinformatics/btz597)
Supplement: btz597_Supplementary_Material [file btz597_supplementary_material.pdf]

Supplementary Material to *Sequence tube maps: making graph genomes intuitive to commuters*

Guided Walk-Through

Go to the demonstration page: https://vgteam.github.io/sequenceTubeMap/

Control Bar

The control bar contains a variety of control buttons. Their function is described in the sections below.

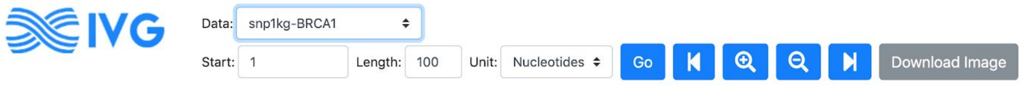

Viewing panel

The default view illustrates a stretch of the BRCA1 gene. We constructed a graph from GRCh38 and known 1000 Genomes variants, then aligned short reads to it.

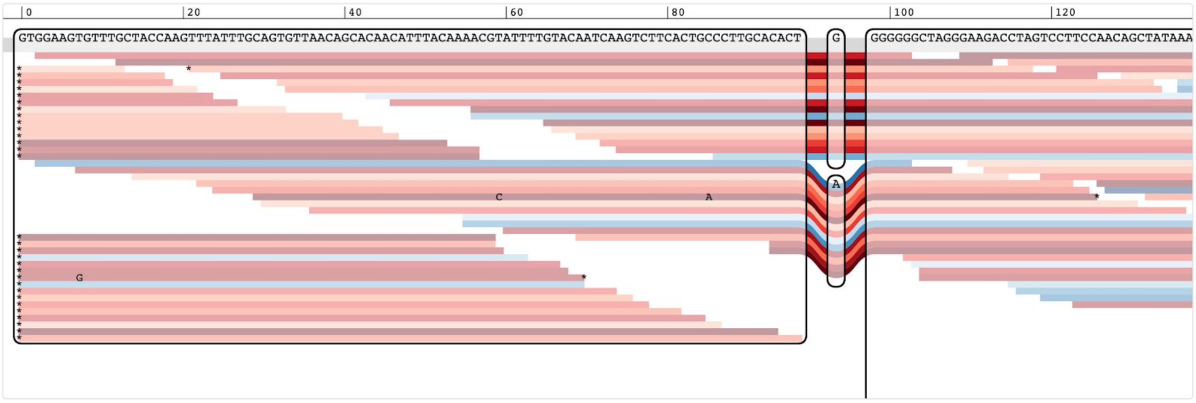

- To change the gene location, you can either:
    - drag this image left and right with the mouse,
    - In the control bar, use the left and right arrows,
    - In the control bar, specify a starting point and image length, and click ‘Go’.
- You can zoom/unzoom:
    - as you normally do on your browser (e.g. trackpad)
    - In the control bar, by clicking on the magnifying glass buttons

- Hovering over a path highlights it unless it is hidden behind a node (see node transparency below).
- There are two types of paths on the graph:
  - The thicker paths are haplotypes (their colors are specifically individually in the legend below). Note that the default dataset (see Changing Datasets below) only has one haplotype, namely a stretch of the reference genome. Assuming there are more than one haplotypes, double clicking on a haplotype rearranges the graph so an to maximise the linearity of that haplotype (in effect it becomes the new reference)
  - The thinner paths are reads: reads are colored dynamically, either by strand (with respect to the view) or by mapping quality. See Visualization Options below to toggle between these different modes.

Configuration menu

Below the display is a configuration menu. To open them up or close them, click on their title bars:

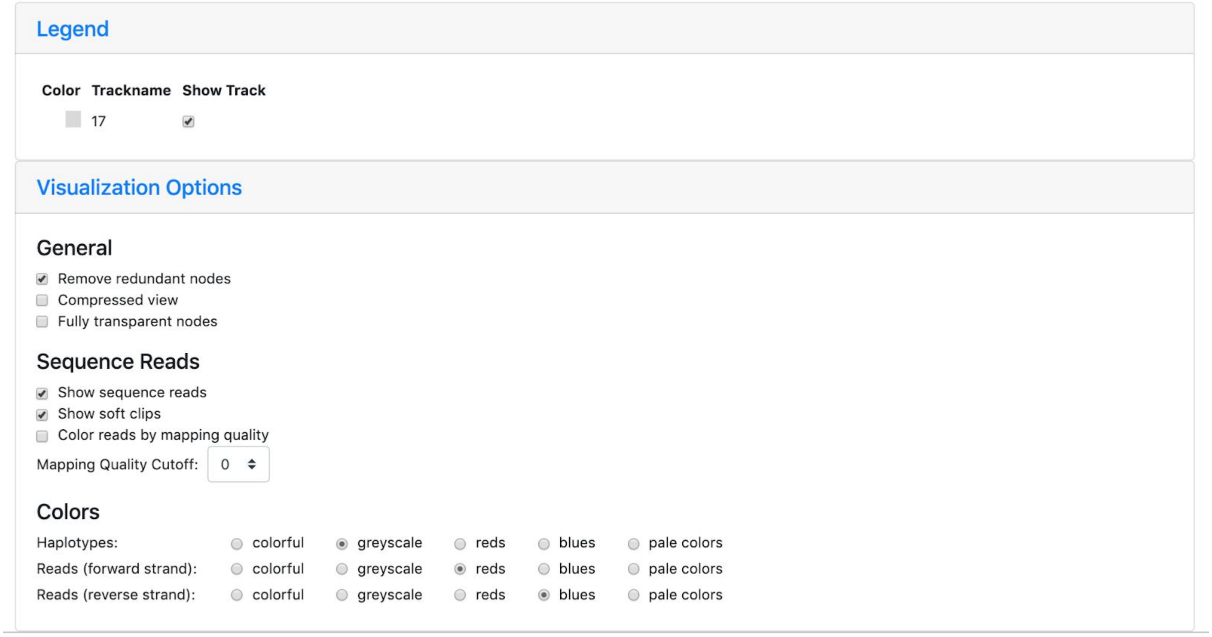

- Legend:
  - This menu toggles the display of specific haplotypes on and off.
- Visualisation Options:
  - General:
    - Remove redundant node: automatically collapses linear chains of nodes
    - Compressed view: compresses the width of the nodes, to display a larger region in the same space.
    - Fully transparent nodes: allows you to hover over individual reads and highlight them.
  - Sequence
    - Show sequence reads

        - Show soft clips
        - Color reads by mapping quality
    - Colors:
        - Haplotypes
        - Reads (forward strand)
        - Reads (negative strand)

Changing datasets

You can change datasets by clicking on the 'Data' drop down menu at the very top of the display, then clicking 'Go' on the Control Bar.

- snp1kg-BRCA1: (default) illustrates a stretch of the BRCA1 gene. We constructed a graph from GRCh38 and known 1000 Genomes variants, then aligned short reads to it (http://public.gi.ucsc.edu/~anovak/hgvm/BRCA1/)
- cactus: the same as above, but produced using Cactus (https://github.com/ComparativeGenomicsToolkit/cactus)
- vg "small" example: artificial example used for testing vg
- Synthetic data examples: 5 types of common genomic rearrangements and a use case are displayed on artificial examples:
    - Indels and Polymorphisms only
    - Inversions
    - Nested Inversions
    - Duplications
    - Translocations
    - Aligned Reads
- Custom (file upload)
    - This mode requires the user to upload an xg, gbwt and gam file (see https://github.com/vgteam/vg)
- Custom (mounted file)
    - This mode allows to you use example *vg* files, via drop down menus.
